# *Inquilinus limosus* Bacteremia in Lung Transplant Recipient after SARS-CoV-2 Infection

**DOI:** 10.3201/eid2903.221564

**Published:** 2023-03

**Authors:** Eric Farfour, Mathilde Zrounba, Antoine Roux, Hélène Revillet, Alexandre Vallée, Marc Vasse

**Affiliations:** Hôpital Foch, Suresnes, France (E. Farfour, M. Zrounba, A. Roux, A. Vallée, M. Vasse);; CHU de Toulouse, Toulouse, France (H. Revillet);; Observatoire National *Burkholderia cepacia*, Toulouse (H. Revillet);; UMRS 1176, le Kremlin-Bicêtre, Paris-Saclay, France (M. Vasse)

**Keywords:** *Inquilinus limosus*, COVID-19, SARS-CoV-2, bacteria, bacteremia, graft dysfunction, lung transplant recipient, cystic fibrosis, France, viruses, respiratory infections, *Suggested citation for this article*: Farfour E, Zrounba M, Roux A, Revillet H, Vallée A, Vasse M. *Inquilinus limosus* bacteremia in lung transplant recipient after SARS-CoV-2 infection. Emerg Infect Dis. 2023 Mar [*date cited*]. https://doi.org/10.3201/eid2903.221564

## Abstract

*Inquilinus limosus* is an environmental bacterium associated with respiratory tract colonization in cystic fibrosis patients. We report a case of *I. limosus* bacteremia in a patient in France who received a lung transplant and experienced chronic graft dysfunction and SARS-CoV-2 infection. This case suggests *I. limosus* displays virulence factors associated with invasion.

A 45-year-old woman in France who had received a lung transplant in 2016 for end-stage cystic fibrosis (CF) sought care for rhinorrhea on March 1, 2022. Her immunosuppressive regimen included mycophenolate mofetil (750 mg 2×/d) and cyclosporine A (200 mg 2×/d). She received oral azithromycin (250 mg/d) and trimethoprim/sulfamethoxazole 400 (80 mg/d) for pneumocystosis prophylaxis. In 2021, she experienced progressive graft dysfunction with no obvious trigger and was treated with alemtuzumab in June 2021. Her forced expiratory volume decreased from 78% in January 2021 to 57% in May 2021 and 30% in January 2022.

At the visit, the patient tested positive for SARS-CoV-2 by reverse transcription PCR. She returned home with treatment for symptoms; she had a productive cough with greenish sputum, for which she received 7 days of amoxicillin/clavulanate. Because her condition did not improve, she continued the treatment for 14 more days. On April 11, we isolated a strain of *P. aeruginosa* (10^6^ CFU/mL) from her sputum and prescribed cefepime (2 g/d). However, her condition worsened, and she was hospitalized on April 20. A chest computed tomography showed bilateral nodular condensations in the lungs; we switched cefepime for piperacillin/tazobactam and tobramycin. Several respiratory samples grew *Inquilinus limosus* and *Pseudomonas aeruginosa* (Table). We recovered *A. fumigatus* from bronchoalveolar liquid and started the patient on posaconasole (300 mg/d). No mycobacteria were recovered. 

**Table:** Timeline of events in study of *Inquilinus limosus* bacteremia in lung transplant recipient with history of chronic graft dysfunction and prolonged SARS-CoV-2 infection, France, 2022*

Date	Symptoms and clinical conditions	CT scan findings	Microbiologic findings	Treatment
Mar 1	Rhinorrhea		SARS-CoV-2 RT-PCR positive (Ct = 18.5)	Symptomatic treatment
Mar 14	Productive cough with greenish sputum		SARS-CoV-2 RT-PCR positive (Ct = 17.0)	Amoxicillin/clavulanate 1.5 g/d for 7 d
Mar 29	No improvement			Amoxicillin/clavulanate 1.5 g/d continued for 14 d; oseltamivir added for 5 d
Apr 11	No improvement	Bilateral nodular opacities	Sputum grew 10^6^ CFU/mL of *Pseudomonas aeruginosa,* resistant to ticarcillin, piperacillin, and carbapenem and susceptible to ceftazidime, cefepime, tobramycin, and ciprofloxacine (EUCAST 2021 guidelines); SARS-CoV-2 RT-PCR positive (Ct = 23.2)	Cefepime 2 g/d for 11 d
Apr 22	Condition worsened; patient hospitalized	Discordant evolution with reduction of some lesions but appearance of new condensations and ground-glass opacities	SARS-CoV-2 RT-PCR positive (Ct = 26.4)	Piperacillin/tazobactam 8 g/d for 5 d; tobramycin 5 mg/kg, 1 shot
Apr 23			Sputa grew 10^6^ CFU/mL of *I. limosus* and 10^3^ CFU/mL of a cephalosporin-susceptible *P. aeruginosa*	
Apr 24			Sputa grew 10^6^ CFU/mL of *I. limosus* and 10^3^ CFU/mL a cephalosporin-susceptible *P. aeruginosa*	
Apr 25			BAL grew 10^4^ CFU/mL of *I. limosus*, 10^2^ CFU/mL, cephalosporin-susceptible *P. aeruginosa,* and few colonies of *Aspergillus fumigatus*	
Apr 26	Persistent cough and colored sputum		SARS-CoV-2 RT-PCR positive (Ct = 23.1)	Ceftolozane/tazobactam 0.75 g d
Apr 29			Sputa grew 10^6^ CFU/mL of *I. limosus* and 10^5^ CFU/mL of *P. aeruginosa*	
Apr 30	Fever, no respiratory improvement		1 vial of a blood culture set sampled on April 26 was positive for a gram-negative rod after 87 h of incubation	Posaconazole 300 mg/d
May 2	Apyrexia			Meropenem 2 g/d for 2 d; amikacin 0.75 g, 1 shot
May 3			Gram-negative rod isolated from the blood culture identified as *I. limosus*; antimicrobial susceptibility testing results	
May 4		Increased ground-glass opacities and bilateral condensations		Ciproflxoacin 1 g/d
May 5			SARS-CoV-2 RT-PCR positive (Ct = 27.6); BAL grew 10^4^ CFU/mL of *I. limosus*	
May 12	Respiratory improvement		SARS-CoV-2 RT-PCR positive (Ct = 29.8)	
July 21			Sputa grew 10^6^ CFU/mL of *I. limosus* and 10^6^ CFU/mL of *P. aeruginosa*	

*Blank cells indicate no report. CT, computed tomography; Ct, cycle threshold; EUCAST, European Committee on Antimicrobial Susceptibility Testing; RT-PCR, reverse transcription PCR.

An aerobic vial of a blood culture set sampled on April 26 was positive for *I. limosus* after 87 hours of incubation. We identified *I. limosus* using matrix-assisted laser desorption/ionization time-of-flight mass spectrometry after subculture and formic acid extraction. We replaced the treatment with ceftolozane/tazobactam. We determined MIC as follows: piperacillin/tazobactam, >32 mg/L; cefepime, 256 mg/L; ceftolozane/tazobactam, 256 mg/L; imipenem, 0.047 mg/L; meropenem, 0.012 mg/L; and ciprofloxacin, 0.016 mg/L. Ceftolozane/tazobactam was switched for meropenem plus amikacin and then ciprofloxacin on May 4. The patient improved but remained colonized with *I. limosus*, displaying a similar pattern of resistance, 3 months later. Of interest, she was colonized before the lung transplantation, but *I. limosus* has not been isolated since then.

*I. limosus* is a fastidious, gram-negative rod from environmental sources (*1*) that has rarely been associated with colonization of the respiratory tract of CF patients (*2*). The airways of CF are susceptible to colonization by respiratory pathogens (*3*), a condition that is improved in lung transplant recipients. Nevertheless, *I. limosus* infection has been reported twice in lung transplant recipients (*4*,*5*). In 1 case, a 22 year-old woman had pulmonary infiltrates develop within a month after lung transplantation (*4*). She completely recovered with antimicrobial drug treatment; *I. limosus* was not isolated during 1 year of follow-up. In the second case, a 31-year-old man experienced a bacteremic lung empyema 1 month posttransplant and a contralateral lung empyema 7 months later (*5*). He recovered from each episode with surgery and antimicrobial treatment with ciprofloxacin and meropenem. Both patients were lung transplant recipients for end-stage cystic fibrosis (CF); they were colonized with *I. limosus* before lung transplantation. Indeed, the lung graft microbiome is affected by donor and recipient factors (*6*), but early posttransplant infections mainly involve the bacteria of the recipient rather than those of the donor (*7*). 

In contrast to those patients, the case-patient we describe experienced a late infection several years after *I. limosus* clearance. Unfortunately, the pretransplant strain was not preserved, and we could not determine whether she was infected with the strain she was colonized with before the lung transplantation or another strain. It is possible that the chronic graft dysfunction, the recent intensification of immunosuppression, and the SARS-CoV-2 infection could have led to a modification of the graft microbiome and enabled colonization with *I. limosus*. In CF patients, colonization with *I. limosus* induces a specific serum antibody response (*8*). The intensification of immunosuppression and the clearance of *I. limosus* after lung transplantation could have reduced humoral immunity. Furthermore, the bacteremia suggested virulence factors involved in the invasion. Two other cases of *I. limosus* bacteremia have been reported previously (*5*,*9*).

Because *I. limosus* is a rarely encountered microorganism and because its colonies are of a mucoid morphotype, it could be misidentified using phenotypic characteristics as *P. aeruginosa* (*8*,*10*). The biochemical methods used previously provided inconsistent identification, and neither European Committee on Antimicrobial Susceptibility Testing nor Clinical and Laboratory Standards Institutes guidelines include standardized *I. limosus* antimicrobial susceptibility testing. However, matrix-assisted laser desorption/ionization time-of-flight mass spectrometry accurately identifies *I. limosus*. *I. limosus* displays high MICs for colistin and almost all β-lactams, except imipenem and meropenem (*9*). It has been suggested that the multidrug resistance of *I. limosus* enhances its selection in CF patients (*2*). In our case, successive treatment with drugs that were ineffective against *I. limosus* could have enabled its selection.

In conclusion, we emphasize a pathogenic role of *I. limosus* in lung transplant recipients several years after respiratory clearance of the bacteria. Chronic graft dysfunction, intensifying immunosuppression, and SARS-CoV-2 infection in this patient could have favored colonization with *I. limosus*. Characteristics of the bacterium such as colony morphotypes and multidrug resistance could delay effective therapy.
